# Automated detection of lung cancer-caused metastasis by classifying scintigraphic images using convolutional neural network with residual connection and hybrid attention mechanism

**DOI:** 10.1186/s13244-022-01162-2

**Published:** 2022-02-09

**Authors:** Yanru Guo, Qiang Lin, Shaofang Zhao, Tongtong Li, Yongchun Cao, Zhengxing Man, Xianwu Zeng

**Affiliations:** 1 School of Mathematics and Computer Science, Northwest Minzu University, Lanzhou, Gansu China; 2 Key Laboratory of Streaming Data Computing Technologies and Application, Northwest Minzu University, Lanzhou, Gansu China; 3 Key Laboratory of China’s Ethnic Languages and Information Technology of Ministry of Education, Northwest Minzu University, Lanzhou, Gansu China; 4 Department of Nuclear Medicine, Gansu Provincial Tumor Hospital, Lanzhou, Gansu China

**Keywords:** Bone scan, Skeletal metastasis, Lung cancer, Image classification, Convolutional neural network

## Abstract

**Background:**

Whole-body bone scan is the widely used tool for surveying bone metastases caused by various primary solid tumors including lung cancer. Scintigraphic images are characterized by low specificity, bringing a significant challenge to manual analysis of images by nuclear medicine physicians. Convolutional neural network can be used to develop automated classification of images by automatically extracting hierarchal features and classifying high-level features into classes.

**Results:**

Using convolutional neural network, a multi-class classification model has been developed to detect skeletal metastasis caused by lung cancer using clinical whole-body scintigraphic images. The proposed method consisted of image aggregation, hierarchal feature extraction, and high-level feature classification. Experimental evaluations on a set of clinical scintigraphic images have shown that the proposed multi-class classification network is workable for automated detection of lung cancer-caused metastasis, with achieving average scores of 0.7782, 0.7799, 0.7823, 0.7764, and 0.8364 for accuracy, precision, recall, F-1 score, and AUC value, respectively.

**Conclusions:**

The proposed multi-class classification model can not only predict whether an image contains lung cancer-caused metastasis, but also differentiate between subclasses of lung cancer (i.e., adenocarcinoma and non-adenocarcinoma). On the context of two-class (i.e., the metastatic and non-metastatic) classification, the proposed model obtained a higher score of 0.8310 for accuracy metric.

## Key points

- Automated detection of lung cancer-caused skeletal metastasis is first studied.
- Convolutional neural network is exploited to develop automated classification method.
- Clinical scintigraphic images are used to experimentally evaluate the proposed classification model.

## Background

Skeletal metastasis is common in several of prevalent cancers including prostate, breast, and lung cancers [1], with 80% of all skeletal metastatic lesions originating from one of these primary sites [2]. The percentage of metastasis-related death reaches up to 90% for all lung cancer mortality [3]. Early detection of skeletal metastasis is extremely important not only for decreasing morbidity but also for disease staging, outcome prediction, and treatment planning [4].

Skeletal scintigraphy (bone scan) and positron emission tomography (PET) are commonly used for surveying bone metastasis [5, 6]. Compared to PET, bone scan is more affordable and available due to its low-cost equipment and radiopharmaceutical. Bone scan is typically characterized by high sensitivity but low specificity, bringing significant challenge to manual analysis of bone scan images by nuclear medicine physicians. The reasons of low specificity are multi-fold, mainly including low spatial resolution, accumulation of radiopharmaceutical in normal skeletal structures, soft tissues or viscera, and uptake in benign processes [7].

Automated analysis of bone scan images becomes therefore desired for accurate diagnosis of skeletal metastasis. There has been a substantial amount of works aimed at developing automated diagnosis approaches using conventional machine learning models to classify bone scan images into classes [5, 8–11], where the image features were manually extracted by researchers. The handcrafted features, however, often suffer from insufficient capability and unsatisfied performance for clinical tasks [6].

Convolutional neural network (CNN), a mainstream branch of deep learning techniques, has gained huge success in automated analysis of natural images [12–14] and medical images [14–17] due to their ability to automatically extracting hierarchical features from images in an optimal way. CNN-based automated classification methods have been proposed to detect metastasis caused by a variety of various primary tumors including prostate cancer [18–23], breast cancer [22–24], lung cancer [25, 26], and both of them [25–27]. The main purpose of existing works is to develop two-class classification models to determine whether or not an image contains metastasized lesion(s) by classifying this image (normal and metastatic). Differently, a series of CNN-based methods has been proposed to classify whole-body scintigraphic images for automated detection of skeletal metastases in our previous works [28, 29], in which we did not distinguish between the primary cancers.

Targeting at automated detection of skeletal metastasis caused by lung cancer, in this work, we propose a CNN-based multiclass classification network to classify whole-body scintigraphic images acquired from patients with clinically diagnosed lung cancer using a SPECT (single photon emission computed tomography) imaging device (i.e., GE SPECT Millennium MPR). The proposed network can not only determine whether an image contains lung cancer-caused skeletal metastasis, but also differentiate between subclasses of lung cancer (i.e., adenocarcinoma and non-adenocarcinoma).

The main contributions of this work can be summarized as: First, to the best of our knowledge, we are the first to attempt to solve the problem of automated detection of skeletal metastasis originated from various subclasses of lung cancer. Second, we convert the detection problem into the multiclass classification of low-resolution, large-size scintigraphic images using a CNN-based end-to-end network that first extracts hierarchal features from images, then aggregates these features, and finally classifies those high-level features into classes. Lastly, we use a group of scintigraphic images acquired from patients with clinically diagnosed lung cancer to evaluate the proposed method. Experimental results have shown that our CNN-based classification network performs well for distinguishing SPECT images between non-metastatic and metastatic as well as their sub-classes of metastasis.

The rest of this paper is organized as follows. We present in “Methods” section the proposed method. We report in “Results” section the experimental evaluation conducted on clinical SPECT images. In “Discussion” secton, we provide a brief discussion on the reasons that cause the misclassifications. In “Conclusions” section, we conclude this work and point out the future research directions.

## Methods

**Fig. 1:**
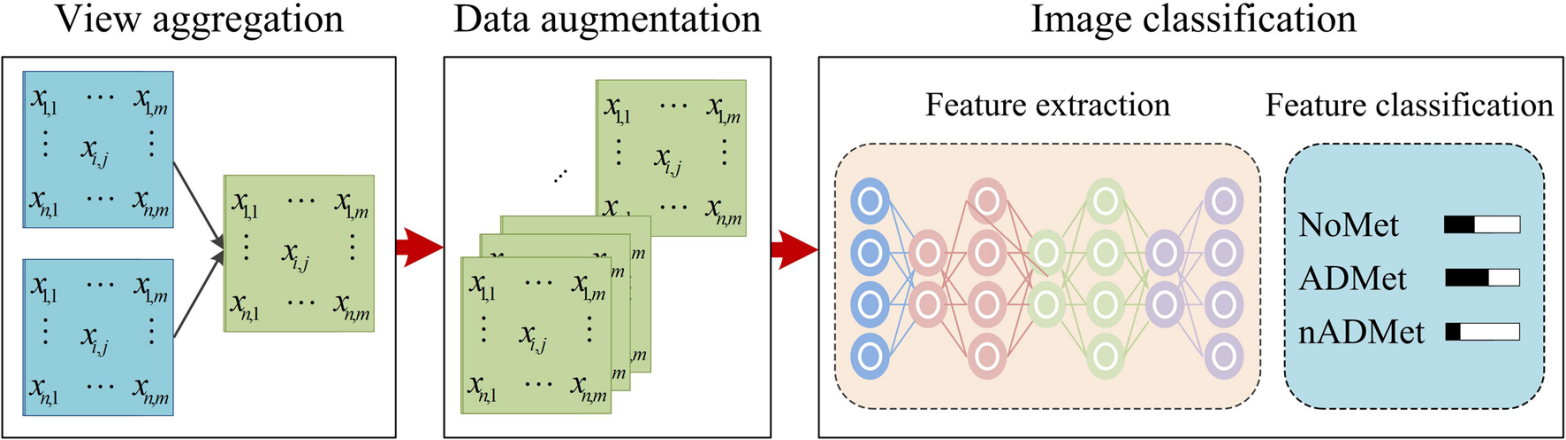
Overview of the proposed CNN-based multiclass classification method

To automatically detect metastasis of lung cancer in scintigraphic images, image fusion operation is first employed to enhance the lesion(s) in low-resolution whole-body scintigraphic images through aggregating the anterior- and posterior-view images of each bone scan. Parametric variation-based data augmentation is then applied to expand the size of the dataset used in this work to improve the performance of CNN-based network on classifying images as much as possible. A CNN-based end-to-end network is developed to classify the fused images by first extracting hierarchal features from images, then aggregating features, and finally classifying high-level features into classes of concerns, i.e., without metastasis (NoMet), adenocarcinoma metastasis (ADMet) and non-adenocarcinoma metastasis (nADMet). Figure 1 provides an overview of the proposed multiclass classification method, comprising of three main steps, i.e., view aggregation, data augmentation, and image classification.

### View aggregation

**Fig. 2:**
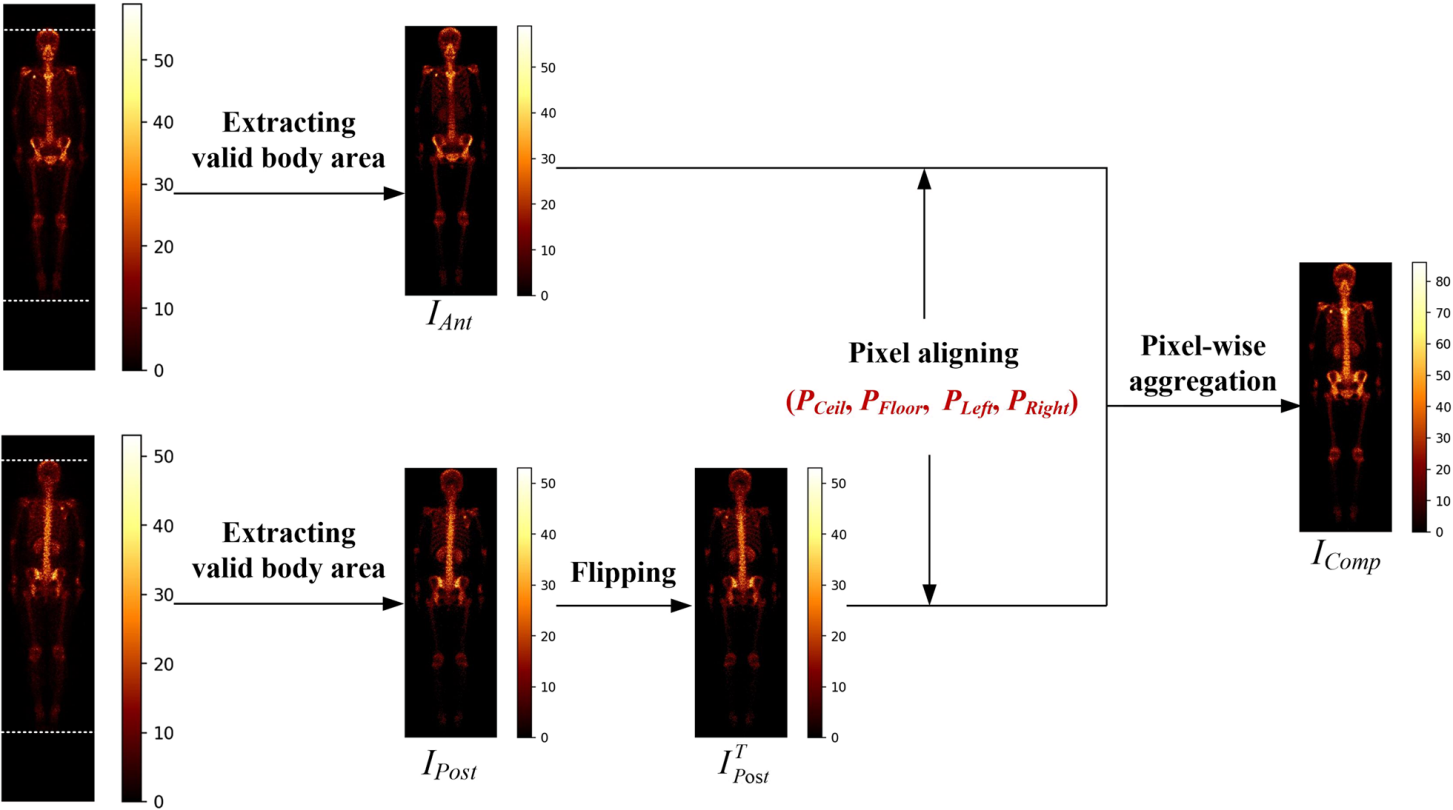
Illustration of view aggregation for enhancing metastatic lesions

During the SPECT imaging, two whole-body images were collected for each patient, corresponding to the anterior- and posteriorviews, respectively. When a primary tumor (e.g., lung cancer) invades into bone tissue, there will be an area of increased radionuclide’s uptake in the image. It is common, however, the metastatic areas have varied intensity of uptake in anterior- and posterior-view images. How to enhance the metastatic areas in images becomes crucial for accurate detection of metastasis. A pixel-wise view aggregation method is proposed to ‘excite’ those metastatic pixels, while ‘squeeze’ the normal pixels by fusing two views as shown in Fig. 2.

Let *I*_Ant_ and *I*_Post_ denote the anterior- and posterior-view image respectively, the pixel-wise view aggregation method works as follows.

#### Image flipping

The posterior-view image *I*_Post_ is flipped horizontally around its central vertical line to obtain an image *IT *Post.

#### Pixel aligning

A horizontal line sweeps the image (i.e., *I*_Ant_ and *IT *Post) line by line to find out the critical points *P*_Ceil_ and *P*_Floor_ by examining the pixel value that represents uptake intensity. Similarly, we use a vertical line to sweep image line by line to find out the critical points *P*_Left_ and *P*_Right_. Two images *I*_Ant_ and *I*_Post_^T^ will be aligned according to these four critical points.

#### Pixel-wise image addition

The aligned images, *I*_Ant_ and *I*_Post_^T^, will be aggregated to generate a composite image, *I*_Comp_, according to Eq. 1.

1 $$I_{{{\text{Comp}}}} = f\left( {I_{{{\text{Ant}}}} ,I_{{{\text{Post}}}}^{T} } \right)$$

where *f* is aggregation function, i.e., pixel-wise addition operation.

### Data augmentation

It is widely accepted that the classification performance of CNN-based models depends on the size of dataset, with high classification accuracy always corresponding to the large dataset. Currently, a variety of various methods can be utilized to augment dataset including the parametric variation and adversarial learning techniques. In this work, we use the parametric variation technique to augment our dataset since the parametric variation-based data augmentation can obtain samples that have the same distribution as the original ones with the lower time complexity. Specifically, image translation and rotation are used, which are detailed as follows [30].

#### Image rotation

**Fig. 3:**
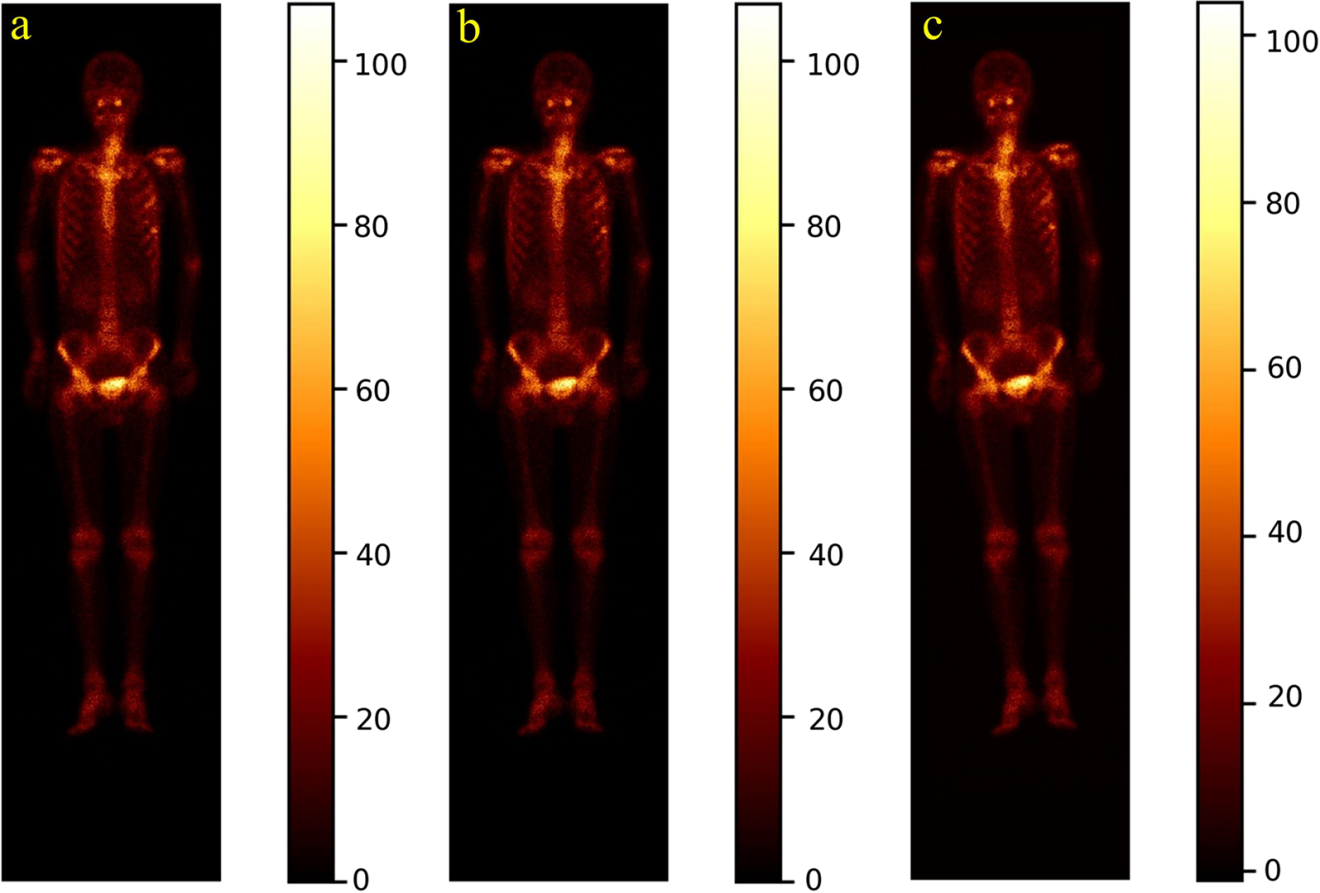
Illustration of translating and rotating whole-body SPECT scintigraphic image. **a** Original posterior image; **b** Translated image; and (**c**) rotated image by 3° to the left direction

Given a constant *r* ∈ [0, *r*_*T*_], an image will be randomly rotated by *r*^o^ in either the left or right direction around its geometric center. The parameter *r*_*T*_ is experimentally determined according to the distribution of the radiotracer uptake of all images in the dataset. Figure 3d depicts the obtained image by rotating the image in Fig. 3a to the right direction by 3°.

#### Image translation

Given a constant *r* ∈ [0, *t*_*T*_], an image will be randomly translated by + *t* or −*t* pixels in either the horizontal or vertical direction. The parameter *t*_*T*_ is experimentally determined according to the distribution of the radiotracer uptake of all images in the dataset. Figure 3c shows a resulting example by translating the given image in Fig. 3a + 3 pixels horizontally.

### CNN-based classification network

Table 1 outlines the structure of the proposed 26-layer CNN-based classification network, consisting of one convolution layer (Conv), one normalization layer (Norm), one pooling layer (Pool), a set of residual convolution layer attached attention (RA-Conv) with varied kernel, one global average pooling layer (GAP), and 1 Softmax layer.

**Table 1:** Network structure of the proposed CNN-based classification model

Layer	Configuration
Conv	7 × 7, 64, Stride = 2
Norm	Batch normalization
Pool	3 × 3 Max pooling, Stride = 2
RA-Conv_2	$$\left[ \begin{gathered} 3 \times 3,\;\;64 \hfill \\ 3 \times 3,\;\;64 \hfill \\ \end{gathered} \right] \times 2$$
RA-Conv_3	$$\left[ \begin{gathered} 3 \times 3,\;128 \hfill \\ 3 \times 3,\;128 \hfill \\ \end{gathered} \right] \times 3$$
RA-Conv_5	$$\left[ \begin{gathered} 3 \times 3,\;\;256 \hfill \\ 3 \times 3,\;\;256 \hfill \\ \end{gathered} \right] \times 5$$
RA-Conv_2	$$\left[ \begin{gathered} 3 \times 3,\;\;512 \hfill \\ 3 \times 3,\;\;512 \hfill \\ \end{gathered} \right] \times 2$$
Global average pooling (GAP)	
Softmax	

An input 256 × 1024 scintigraphic image is convolved by the Conv layer with filter of 7 × 7 to calculate a feature map made of neurons, followed by a batch normalization layer and a max pooling layer with kernel of 3 × 3. The subsequent convolutional layers are organized as residual convolution with hybrid attention inside or outside of the convolution. A global average pooling layer is used to alleviate the over-fitting problem while speeding up the training process. The Softmax layer points out the class of an image with a real number. The main layers will be detailed as follows.

#### Normalization layer

Batch normalization [31] is used to accelerate the network training by making normalization a part of the model architecture and performing the normalization for each training mini-batch. With batch normalization, we can thus use much higher learning rates and be less careful about initialization.

#### RA-Conv layer

**Fig. 4:**
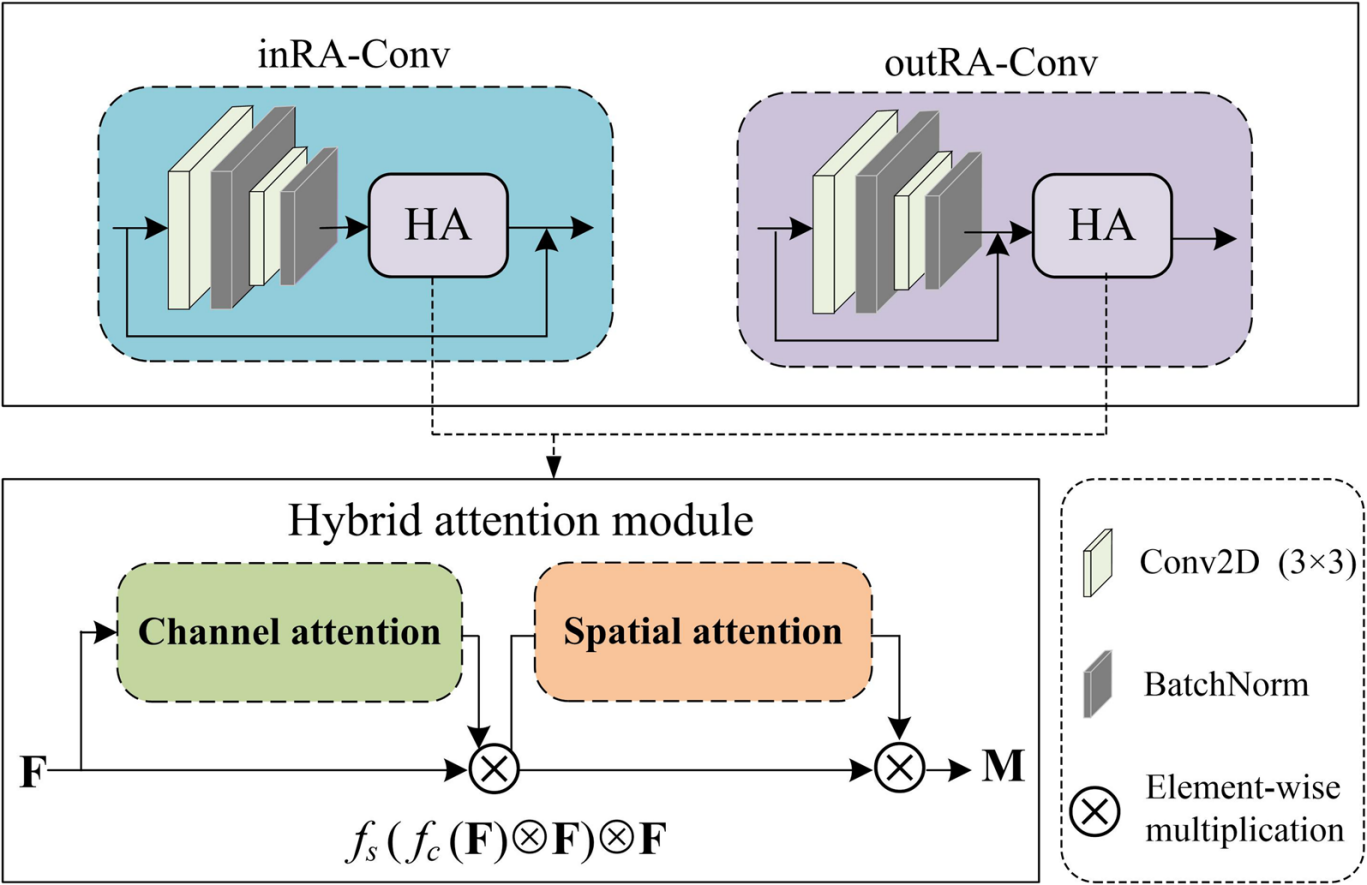
Structure of residual convolution with hybrid attention mechanism

Figure 4 demonstrates the structure of residual convolution with hybrid attention mechanism. We use residual connection to reduce the training parameters and training time. We also introduce hybrid attention mechanism to improve network focusing on those more important areas (i.e., lesions) on the feature maps by considering only the important information. Specifically, we use inRA-Conv (outRA-Conv) to indicate that a hybrid attention module is located inside (outside) the residual convolution. The classifiers are accordingly named as Classifer-inRAC and Classifer-outRAC, respectively.

The cascaded hybrid attention module in Fig. 4 using channel and spatial attention mechanism is capable of computing complementary attention by focusing on ‘what’ (channel attention) and ‘where’ (spatial attention), respectively [32]. Specifically, let **F** be the input of a 2D feature map to the channel attention sub-module. We can obtain a 1D output F, which will be further processed by the spatial attention sub-module to output a refined 2D feature map **M** according to Eq. 2.

2 $${\mathbf{M}} = f_{S} \left( {f_{C} ({\mathbf{F}}) \otimes {\mathbf{F}}} \right) \otimes {\mathbf{F}},$$

where ⊗ is the element-wise multiplication, and *f*_*C*_ and *f*_*S*_ denotes the channel and spatial function, respectively, which are given in Eqs. 3 and 4.

3 $$f_{C} ({\mathbf{F}}) = \sigma \left( {MLP\left( {AvgPool({\mathbf{F}})} \right) + MLP\left( {MaxPool({\mathbf{F}})} \right)} \right),$$

4 $$f_{S} ({\text{F}}) = \sigma \left( {f^{k \times k} \left( {\left[ {AvgPool({\text{F}});MaxPool({\text{F}})} \right]} \right)} \right),$$

where *σ* is the sigmoid function, *MLP* is the multi-layer perceptron, *AvgPool* (*MaxPool*) is the average (max) pooling, and *f*^*k*×*k*^ is a convolutional operation with the kernel size of *k* × *k*.

#### Softmax layer

The network output nodes apply the Softmax function for the number of the unordered classes. A Softmax function is defined in Eq. 5 [33].

5 $$f(x_{j} ) = \frac{{e^{{x_{j} }} }}{{\sum\nolimits_{i = 1}^{n} {e^{{x_{i} }} } }},$$

where *f* (*x*_*j*_) is the score of the *j*-th output node, *x*_*j*_ is the network input to *j*-th output node, and *n* is the number of output nodes. In fact, all of the output values *f* (*x*) are a probability between 0 and 1, and their sum is 1.

## Results

In this section, we provide an experimental evaluation of the proposed network using a set of clinical whole-body scintigraphic images.

### Dataset

In this retrospective study, the whole-body scintigraphic images were collected from the Department of Nuclear Medicine, Gansu Provincial Tumor Hospital from Jan 2014 to Dec 2019 using a single-head gamma camera (GE SPECT Millennium MPR). SPECT imaging was performed between 2 and 3 h after intravenous injection of ^99m^Tc-MDP (20–25 mCi) using a parallel-beam low-energy high-resolution (LEHR) collimator (energy peak = 140 keV, intrinsic energy resolution ≤ 9.5%, energy window = 20%, and intrinsic spatial resolution ≤ 6.9 mm). Each SPECT image was stored in a DICOM (Digital Imaging and Communications in Medicine) file with the imaging size of 256 × 1024. Every element in an image is represented by a 16-bit unsigned integer, differing from the natural images in which element ranges from 0 to 255.

**Fig. 5:**
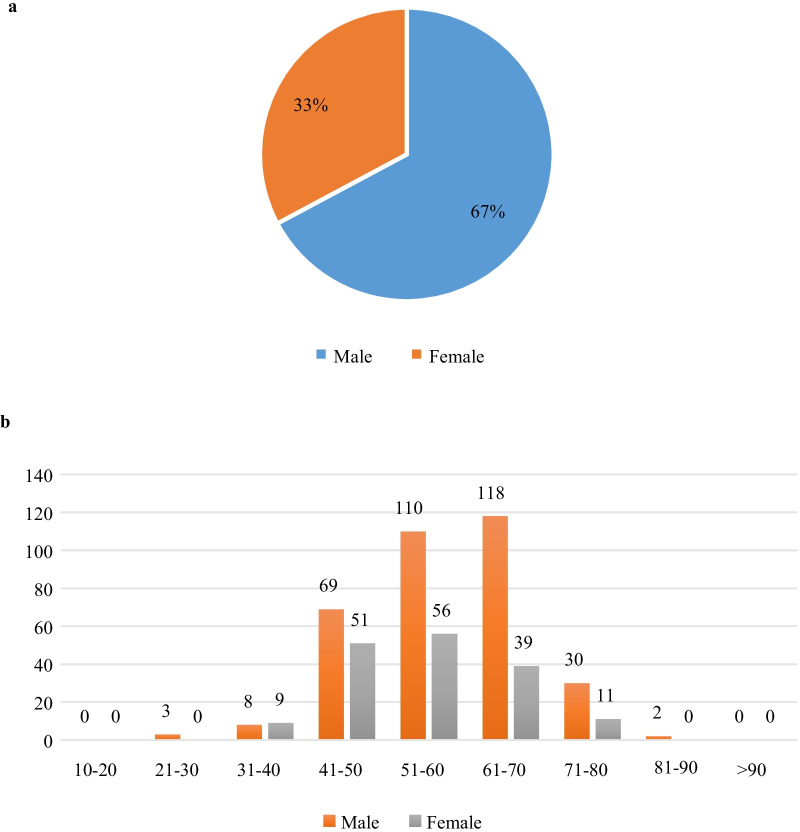
Distribution of patients included in the dataset of whole-body scintigraphic images. **a** Gender; and (**b**) age

A total of 506 patients who were clinically diagnosed with lung cancer were encompassed in this study. Figure 5 demonstrates the distribution of patients with respect to gender and age.

There are 1011 images collected from 506 patients due to the phenomenon of images being not successfully recorded. We categorize all these images into three subclasses, i.e., NoMet (n = 614, ≈ 60.73%), ADMet (n = 237, ≈ 23.44%), and nADMet (n = 160, ≈ 15.83%).

To keep the balance between samples in different subclasses, we randomly selected 226 images from NoMet class and group the original images into dataset D1 as shown in Table 2. Applying data augmentation technique on D1, we obtained an augmented dataset D2. The dataset D3 is achieved by aggregating images in D2.

**Table 2:** An overview of the datasets used in this work

Dataset	ADMet	nADMet	NoMet	Total
D1	237	160	226	623
D2	624	640	614	1878
D3	318	320	307	945

For supervised image classification problem, the CNN-based model is evaluated by comparing the automated classification results against ground truth (human performance) that is often obtained by manually labeling images. However, it is time-consuming, laborious, and subjective to manually label low-resolution, large-size SPECT images. To facilitate labeling SPECT image, in this work, we developed an annotation system based on the LabelMe (http://labelme.csail.mit.edu/Release3.0/) released by MIT.

With LabelMe-based annotation system, imaging findings including the DICOM file and the textual diagnostic report can be imported into the system in advance. In the labeling process, three nuclear medicine physicians from the Department of Nuclear Medicine, Gansu Provincial Tumor Hospital manually labeled areas on the visual presentation of DICOM file with a shape tool (e.g., polygon and rectangle). The labeled area will be annotated with a self-defined code combined with the name of disease or body part. The manually labeled results for all images act as ground truth in the experiments and form an annotation file together to feed into the classifiers.

### Experimental setup

The evaluation metrics we use are *accuracy*, *precision*, *recall*, *specificity*, *F*-1 score, and AUC (Area Under ROC Curve), which are defined in Eqs. 6–10.

6 $${\text{Accuracy}} = \frac{TP + TN}{{TP + TN + FP + FN}},$$

7 $${\text{Precision}} = \frac{TP}{{TP + FP}},$$

8 $${\text{Recall}} = \frac{TP}{{TP + FN}},$$

9 $${\text{Specificity}} = \frac{TN}{{TN + FP}},$$

10 $$F - 1 = 2 \times \frac{{{\text{Precision}} \times {\text{Recall}}}}{{{\text{Precision}} + {\text{Recall}}}},$$

where the notations are *TP* = True Positive, *TN* = True Negative, *FP* = False Positive and *FN* = False Negative.

It is desirable that a classifier shows both a high true positive rate (*TPR* = *Recall*), and a low false positive rate (*FPR* = 1–*Specificity*) simultaneously. The ROC curve shows the true positive rate (*y*-axis) against the false positive rate (*x*-axis), and the AUC value is defined as the area under the ROC curve. As a statistical explanation, the AUC value is equal to the probability that a randomly chosen positive image is ranked higher than a randomly chosen negative image. Therefore, the closer to 1 the AUC value is, the higher performance the classifier achieves.

We divided every dataset (D1, D2 and D3) into two parts: training set and testing set, with the ratio of them being 7: 3. It means that we use 70% of samples in each dataset to train the classifiers, and the rest 30% for testing the classifiers. Images including the augmented ones from the same patient were not divided into the different subsets because they would show similarities. The parameters setting is shown in Table 3.

**Table 3:** Parameters setting of the proposed classification network

Parameter	Value
Learning rate	0.01
Optimizer	Adam
Batch size	32
Epoch	300

The experiments are run in Tensorflow 2.0 on an Intel Core i7-9700 PC with 32 GB RAM running Windows 10.

### Experimental results

For the proposed multiclass classifiers Classifer-inRAC and Classifer-outRAC, Table 4 reports the scores of the defined evaluation metrics obtained on the testing samples in dataset D3.

**Table 4:** Scores of evaluation metrics obtained by Classifer-inRAC and Classifer-outRAC on testing samples in dataset D3

Classifier	Accuracy	Precision	Recall	F-1 score
Classifer-inRAC	**0.7782**	**0.7799**	**0.7823**	**0.7764**
Classifer-outRAC	0.6725	0.7233	0.6831	0.6723

Best value in each column is highlighted in bold

Table 4 shows that the classifier Classifer-inRAC performs better than Classifer-outRAC. Results in Table 5 further show that Classifer-inRAC obtains the best performance on the aggregated samples in augmented dataset (i.e., D3).

**Table 5:** Scores of evaluation metrics obtained by Classifer-inRAC on the testing samples in datasets D1, D2, and D3

Dataset	Accuracy	Precision	Recall	F-1 score
D1	0.6150	0.6324	0.6227	0.6058
D2	0.6968	0.7001	0.7024	0.6930
D3	**0.7782**	**0.7799**	**0.7823**	**0.7764**

Best value in each column is highlighted in bold

**Fig. 6:**
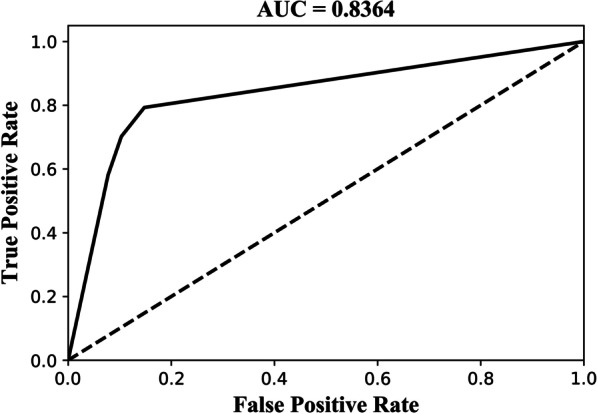
ROC curve and AUC value obtained by Classifer-inRAC on classifying the testing samples in D3

Figure 6 shows the ROC curve and AUC value obtained by Classifer-inRAC on classifying the testing samples in D3, where AUC value = 0.8364.

**Fig. 7:**
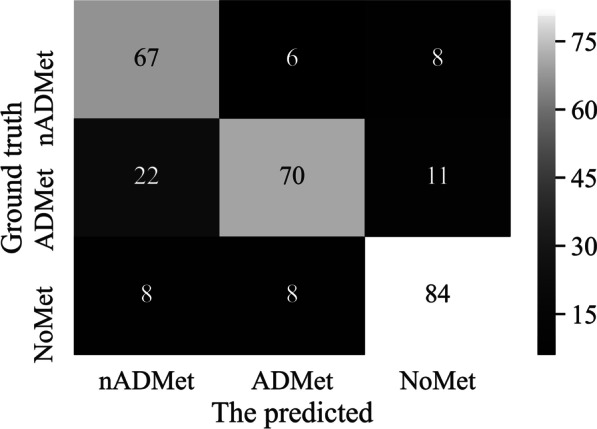
Confusion matrix obtained by Classifer-inRAC on classifying the testing samples in D3

**Fig. 8:**
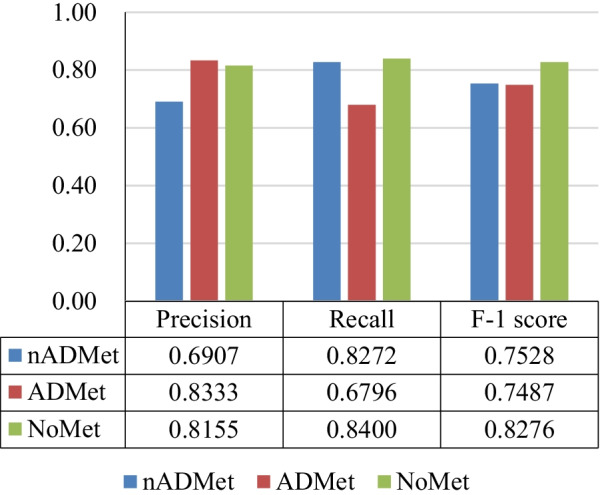
Scores of evaluation metrics obtained by Classifer-inRAC on classifying subclasss on the testing samples in D3

We further examine the ability of Classifer-inRAC on differentiating between the subclasses of images in dataset D3 by providing confusion matrix in Fig. 7 and scores of evaluation metrics in Fig. 8.

Experimental results in Figs. 7 and 8 show that differentiating subclasses between images with metastasis is more challenging than differentiating between metastatic and non-metastatic images. There are 22 ADMet images that have been incorrectly identified as nADMet ones.

With the testing samples in dataset D3, we show the impacts of network structure and depth on classification performance obtained by the proposed classifier Classifer-inRAC.

Table 6 reports the scores of evaluation metrics obtained by Classifer-inRAC after we remove the residual connection and hybrid attention module from Classifer-inRAC.

**Table 6:** Effects of network structure on classification performance obtained on dataset D3

Residual	Attention	Accuracy	Precision	Recall	F-1 score
×	×	0.6937	0.7032	0.7000	0.6940
×	√	0.7042	0.7416	0.7047	0.7031
√	×	0.7500	0.7614	0.7532	0.7497
√	√	**0.7782**	**0.7799**	**0.7823**	**0.7764**

Best value in each column is highlighted in bold

It shows that the best performance can be obtained if Classifer-inRAC has residual connection and hybrid attention module simultaneously from the scores of evaluation metrics as shown in Table 6. Separately, residual connection has more positive impact than hybrid attention mechanism on the classification performance.

Following the architectural design of Classifer-inRAC, we define two classifiers with different network depth, which are given in Table 7.

**Table 7:** Overview of classifiers with similar structure but different depth from Classifer-inRAC

	**Clasifier-18**	**Clasifier-34**	**Clasifier-inRAC**
**Layer**	**Configuration**		
Conv	7 × 7, 64, Stride = 2		
Norm	Batch normalization		
Pool	3 × 3 Max pooling, Stride = 2		
RA-Conv	$$\left[ \begin{gathered} 3 \times 3,\;\;64 \hfill \\ 3 \times 3,\;\;64 \hfill \\ \end{gathered} \right] \times 2$$	$$\left[ \begin{gathered} 3 \times 3,\;\;64 \hfill \\ 3 \times 3,\;\;64 \hfill \\ \end{gathered} \right] \times 3$$	$$\left[ \begin{gathered} 3 \times 3,\;\;64 \hfill \\ 3 \times 3,\;\;64 \hfill \\ \end{gathered} \right] \times 2$$
RA-Conv	$$\left[ {\begin{array}{*{20}c} {3 \times 3,\;\;128} \\ {3 \times 3,\;\;128} \\ \end{array} } \right] \times 2$$	$$\left[ \begin{gathered} 3 \times 3,\;\;128 \hfill \\ 3 \times 3,\;\;128 \hfill \\ \end{gathered} \right] \times 4$$	$$\left[ \begin{gathered} 3 \times 3,\;\;128 \hfill \\ 3 \times 3,\;\;128 \hfill \\ \end{gathered} \right] \times 3$$
RA-Conv	$$\left[ \begin{gathered} 3 \times 3,\;\;256 \hfill \\ 3 \times 3,\;\;256 \hfill \\ \end{gathered} \right] \times 2$$	$$\left[ \begin{gathered} 3 \times 3,\;\;256 \hfill \\ 3 \times 3,\;\;256 \hfill \\ \end{gathered} \right] \times 6$$	$$\left[ \begin{gathered} 3 \times 3,\;\;256 \hfill \\ 3 \times 3,\;\;256 \hfill \\ \end{gathered} \right] \times 5$$
RA-Conv	$$\left[ \begin{gathered} 3 \times 3,\;\;512 \hfill \\ 3 \times 3,\;\;512 \hfill \\ \end{gathered} \right] \times 2$$	$$\left[ \begin{gathered} 3 \times 3,\;\;512 \hfill \\ 3 \times 3,\;\;512 \hfill \\ \end{gathered} \right] \times 3$$	$$\left[ \begin{gathered} 3 \times 3,\;\;512 \hfill \\ 3 \times 3,\;\;512 \hfill \\ \end{gathered} \right] \times 2$$
Global average pooling (GAP)			
Softmax			

**Fig. 9:**
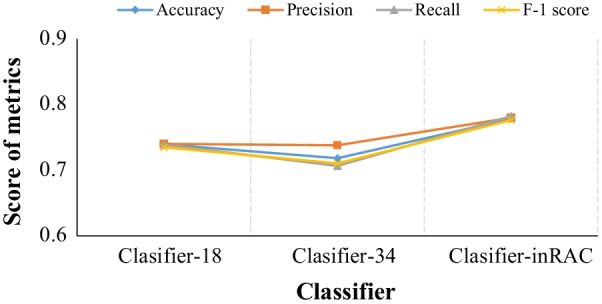
Classfication performance comparison between different classifiers in Table 7

Figure 9 reports the scores of evaluation metrics obtained by the classifiers defined in Table 7 and Classifer-inRAC, showing comparative advantage of the proposed classifier on classifying whole-body images.

**Fig. 10:**
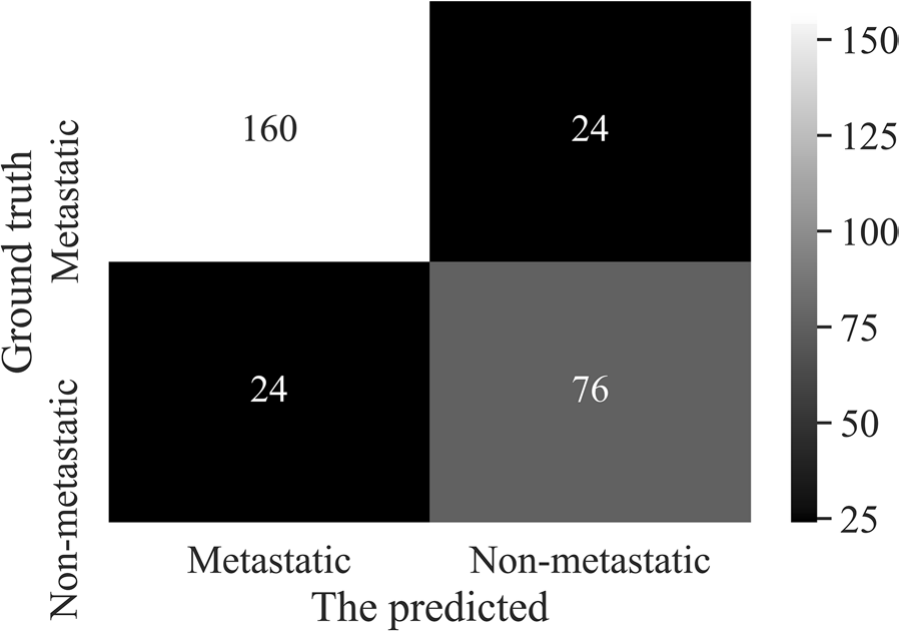
Confusion matrix of two-class classification obtained by Classifer-inRAC

We further test the performance of Classifer-inRAC on two-class classification by merging the metastatic subclasses (i.e., ADMet and nADMet) in the dataset D3. Specifically, the dataset for two-class classification is consisted of metastatic images (*n* = 638, ≈ 67.51%) and non-metastatic images (*n* = 307, ≈ 32.49%). Table 8 reports the scores of evaluation metrics on two-class classification of testing samples and Fig. 10 depicts the corresponding confusion matrix.

**Table 8:** Two-class classification performance obtained by Classifer-inRAC

Accuracy	Precision	Recall	F-1 score	AUC
0.8310	0.8696	0.8696	0.8696	0.8147

The results of two-class classification show that our classifier performs better on differentiating between metastatic and non-metastatic images than classifying images in different subclasses.

A comparable analysis has also been performed between the proposed model and two classical deep models Inception-v1 [34] and VGG 11 [35], which are given in Table 9 by providing their network structures.

**Table 9:** An overview of two classical CNNs-based models used for comparative analysis

Model	Number of weight layers	Filter	Activation	Learning rate
Inception-v1	9 Inception blocks	1 × 1, 3 × 3, 5 × 5	ReLU	10^–2^
VGG 11	11	3 × 3	ReLU	10^–2^

The scores of evaluation metrics obtained by three classifiers on the dataset D3 are reported in Table 10, showing that our model is more suitable for classifying lung cancer-caused images than the classical models. The possible reason is that the network structure of our model (i.e., residual convolution combined with hybrid attention) is capable of extracting more representative features of metastatic lesions.

**Table 10:** Scores of evaluation metrics obtained by the proposed model and two classical models

Model	Accuracy	Precision	Recall	F-1 score
Inception v1	0.5387	0.6003	0.5490	0.5415
VGG 11	0.7324	0.7309	0.7333	0.7309
Classifer-inRAC	**0.7782**	**0.7799**	**0.7823**	**0.7764**

Best value in each column is highlighted in bold

## Discussion

**Fig. 11:**
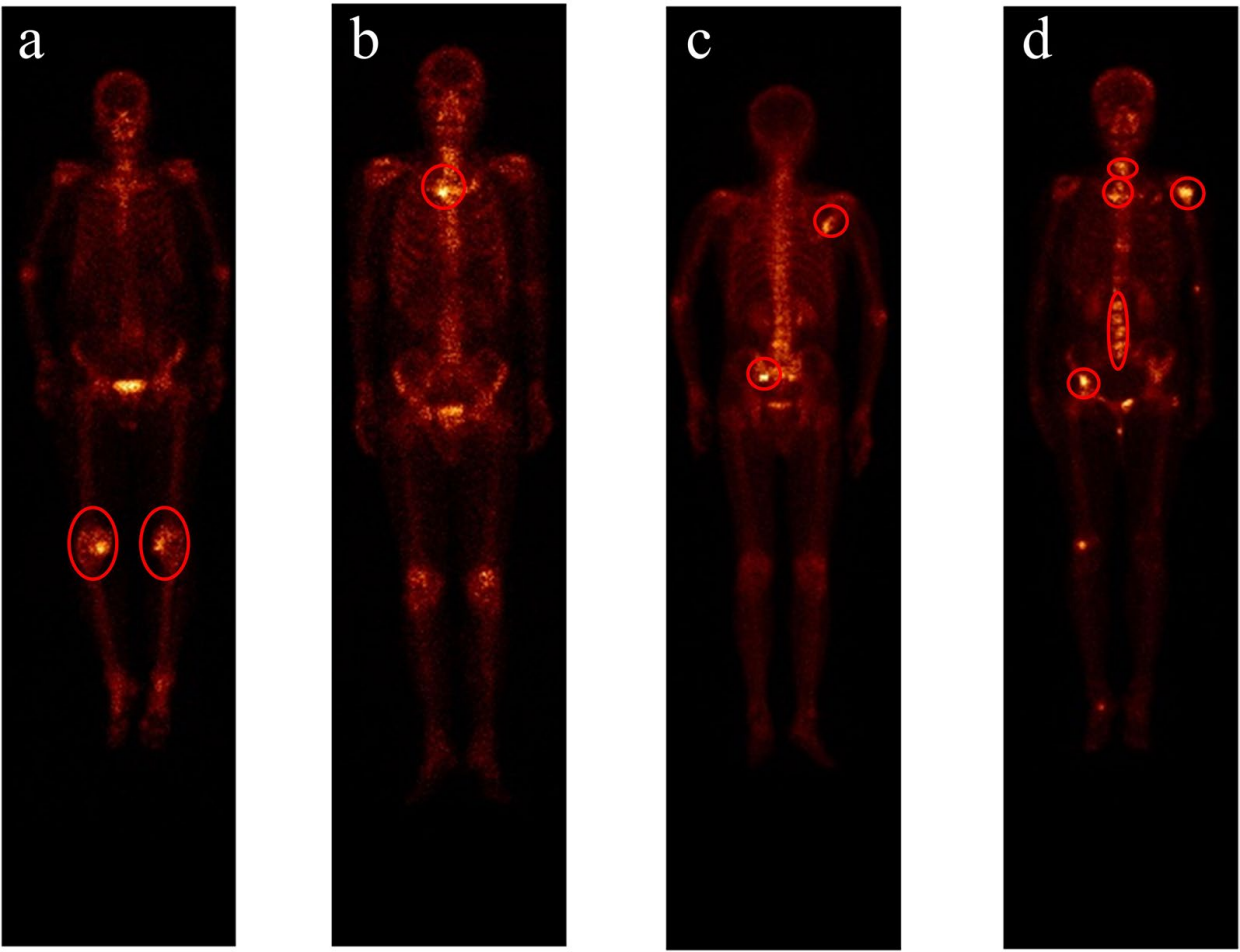
An illustration of the classified images by multiclass classifier Classifer-inRAC. **a** NoMet incorrectly detected as metastatic; **b** ADMet incorrectly detected as nADmet; **c** Correctly detected nADMet image; and (**d**) Correctly detected ADMet image

In this section, we provide a brief discussion about the reasons that may cause the misclassifications by providing a group of examples in Fig. 11.

Now, we provide the reasons for misclassification explained by one nuclear medicine physician and one oncologist from Gansu Provincial Tumor Hospital.

### Misclassification between the metastatic and non-metastatic

Uptake of ^99m^Tc-MDP in benign processes (i.e., knee arthritis) is detected as metastatic lesions by the developed classifier due to the visually similar appearances to skeletal metastasis (see Fig. 11a). Furthermore, a normal bone would show a higher concentration of activity in trabecular bone with a large-mineralizing surface area like the spine. This brings huge challenge to the CNN-based automated classification of SPECT images, hence the metastatic images being misclassified as non-metastatic.

### Misclassification between the diseased subclasses

It is very challenging to accurately classify metastatic images since skeletal metastases are often distributed irregularly in the axial skeleton and typically show variability in size, shape, and intensity [7]. The irregularly distributed radioactivity of ADMet can mimic nADMet, and vice versa, resulting in misclassification between ADMet and nADMet (see Fig. 11b).

### Multiclass classification vs. two-class classification

Multiclass classification aims to not only determine whether an image contains lung cancer-caused skeletal metastasis, but also differentiate between subclasses of lung cancer (i.e., ADMet and nADMet). This is more difficult than to answer that an image whether contains metastasis (i.e., two-class classification). So, the proposed classifier Classifer-inRAC obtained score of 0.8310 and 0.7782 for accuracy metric for multiclass and tow-class classification problems, respectively.

**Fig. 12:**
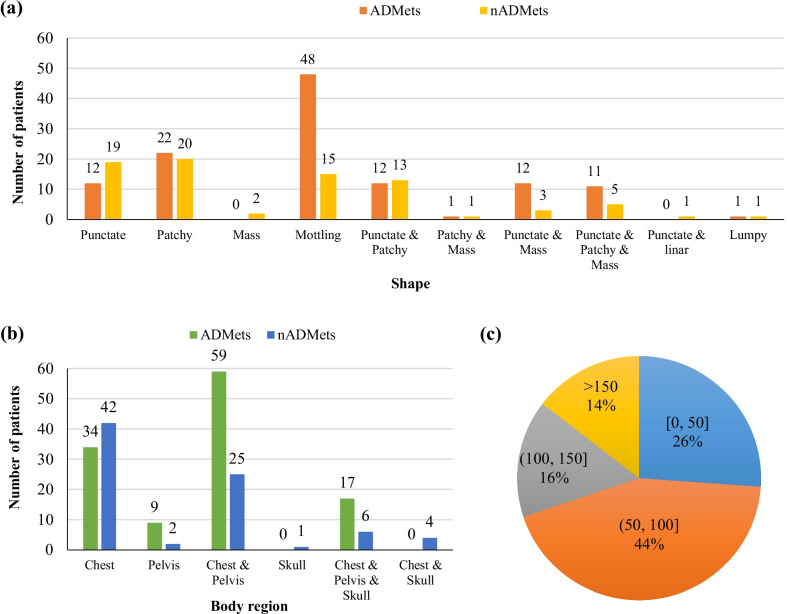
Characteristics of metastatic lesions in ADMet and nADMet subclasses. **a** shape; **b** body region; and (**c**) uptake intensity

Metastatic lesions are further examined by providing statistical analysis of shape, location (body region), and uptake intensity in Fig. 12. The mottling, patchy, punctate lesions dominate both the ADMet and nADMet metastasis as shown in Fig. 12a. The chest (vertebra and ribs) acts the main location (i.e., body region) where the lung cancer-caused metastasis is frequently present in as shown in Fig. 2b. As shown in Fig. 12c, the distribution of detected uptake intensity ranges widely, with 44% of lesions falling into [50, 100]; and much higher uptake can often be detected in the regions of urinary bladder and injection point. This further reveals that it is more difficult to develop an automated method for analyzing scintigraphic images than natural images in which the value of pixel ranges from 0 to 255.

To alleviate the issues mentioned above, technical solutions need to be developed in the future. With a large-scale dataset of SPECT images, representative image features can be extracted for each kind of subclasses by CNN-based end-to-end classifiers. This would contribute to improving the performance of distinguishing between metastatic and non-metastatic images. Moreover, statistical analysis conducted on large-scale SPECT images and pathologic findings would have the potential to develop a multi-modal fusion classifier, enabling to achieve higher classification performance between metastatic images caused by various subclasses of lung cancer.

## Conclusions

Targeting the automated detection of lung cancer-caused metastasis with SPECT scintigraphy, we have developed a convolutional neural network with the hybrid attention mechanism in this work. Parametric variation was first conducted to augment the dataset of original images. An end-to-end CNN-based classification network has been proposed to automatically extract features from images, aggregate features, and classify high-level features into classes. Clinical whole-body scintigraphic images were utilized to evaluate the developed network. Experimental results have demonstrated that our self-defined network performs well in detecting lung cancer-caused metastasis as well as differentiating between subclasses of lung cancer. The analysis has also been conducted to compare the proposed model with other related models. The results reveal that our method can be used for determining whether an image contains lung cancer-caused skeletal metastasis and differentiating between subclasses of lung cancer.

In the future, we plan to extend our work in the following directions. First, we intend to collect more data of images and laboratory findings to improve the proposed multiclass classification model. Hopefully, a robust and effective computer-aided diagnosis system will be developed. Second, we attempt to develop deep learning-based approaches that can classify whole-body SPECT images with multiple lesions from various primary diseases that may present in a single image.

## Data Availability

Anyone can get the validation subset by emailing the corresponding author by stating that the data is used for research purposes only. The whole dataset will be publicly available in the future.

## Abbreviations

99mTc-MDP: 99MTc-methylene diphosphonate
ADMet: Adenocarcinoma metastasis
AUC: Area Under the Curve
CNN(s): Convolutional Neural Network(s)
DICOM: Digital Imaging and Communications in Medicine
nADMet: Non-adenocarcinoma metastasis
NoMet: Without metastasis
PET: Positron Emission Tomography
ReLU: Rectified Linear Unit
ROC: Receiver Operating Characteristic
SPECT: Single Photon Emission Computed Tomography
